# Strong inverse association between physical fitness and overweight in adolescents: a large school-based survey

**DOI:** 10.1186/1479-5868-4-24

**Published:** 2007-06-05

**Authors:** Pascal Bovet, Robert Auguste, Hillary Burdette

**Affiliations:** 1 Unit for Prevention and Control of Cardiovascular Disease, Ministry of Health, Victoria, Republic of Seychelles; 2 Institute of Social and Preventive Medicine (IUMSP), University Hospital Center and University of Lausanne, Bugnon 17, 1005 Lausanne, Switzerland; 3 National Sports Council, Ministry of Arts, Culture and Sports, Victoria, Republic of Seychelles; 4 Department of Pediatrics, University of Pennsylvania School of Medicine, The Children's Hospital of Philadelphia, Room 1578, 34^th ^and Civic Center Blvd, Philadelphia, PA 19104, USA

## Abstract

**Background:**

Studies examining the relationship between physical fitness and obesity in children have had mixed results despite their interrelationship making intuitive sense. We examined the relationship between physical fitness and overweight and obesity in a large sample of adolescents in the Republic of Seychelles (Indian Ocean, African region).

**Methods:**

All students of four grades of all secondary schools performed nine physical fitness tests. These tests assessed agility, strength and endurance, and included the multistage shuttle run, a validated measure of maximal oxygen uptake. Weight and height were measured, body mass index (BMI) calculated, and "overweight" and "obesity" were defined based on the criteria of the International Obesity Task Force. We defined "lean" weight as age- and sex-specific BMI <10^th ^percentile. Age- and sex-specific percentiles for each fitness test were calculated. "Good" performance was defined as a result ≥75^th ^percentile.

**Results:**

Data were available in 2203 boys and 2143 girls from a total of 4599 eligible students aged 12–15 years. The prevalence of overweight (including obesity) was 11.2% (95% confidence interval: 9.9–12.4) in boys and 17.5% (15.9–19.1) in girls. For 7 of the 9 tests, the relationship between BMI and fitness score, as assessed by locally weighted regression, was characterized by a marked inverse J shape. Students with normal body weight achieved "good" performance markedly more often than overweight or obese students on 7 of the 9 tests of fitness and more often than lean children. For example, good performance for the multistage shuttle run was achieved by 25.6% (SE: 2.1) of lean students, 29.6% (0.8) of normal weight students, 7.9% (1.3) of overweight students and 1.2% (0.9) of obese students.

**Conclusion:**

This cross-sectional study shows a strong inverse relationship between fitness and excess body weight in adolescents. Improving fitness in adolescents, likely through increasing physical activity, might need special interventions that are responsive to the ability and needs of overweight children.

## Background

Studies examining the relationship between physical fitness and obesity in children have had mixed results [1-4] despite their interrelationship making intuitive sense. This issue is important since physical fitness may be more predictive of health outcomes than physical activity, and fitness has been associated with improved health outcomes in children. These outcomes include muscular strength, vasculature characteristics or risk factor modification such as blood lipids, insulin or blood pressure [5-7]. In addition, adequate fitness in childhood is likely to carry beneficial biological and behavioral effects into adulthood, such as physically active children are more likely to become physically active adults [8] and physical fitness in children may protect against future cardiovascular disease [9]. The importance of understanding how fitness is related to obesity is further stressed by the recent worldwide trends in increasing obesity and sedentary habits and declining fitness among youth [10].

While physical activity is considered a behavior with a degree of choice on behalf of the individual involved, physical fitness is an attribute, and fitness components include cardio-respiratory endurance, muscle strength and endurance, flexibility, and body composition [11,12]. In particular, maximal oxygen uptake (VO2 max) is considered the gold standard to assess cardiovascular fitness. Assessment of physical fitness requires the conduct of several tests (as compared to questionnaire to assess physical activity), which can partly explain the paucity of studies on fitness in children, particularly in developing countries. The quantitative nature of physical tests underlies that physical fitness can be assessed more reliably and precisely than questionnaire-based physical activity.

In adults both poor physical fitness and physical inactivity are associated with morbidity and mortality [13,14]. From a public health perspective, it is argued that it is preferable to encourage people to become more physically active rather than to become physically fit, since sedentary people will likely achieve the latter if they do the former [15]. However, cardiorespiratory fitness is more strongly predictive of health outcomes than physical activity [15-18] and most analyses have shown a reduction of at least 50% in mortality among highly fit people compared with low-fit people [17].

In this study we examined whether physical fitness was different in children in the lowest or highest weight categories as compared to children with normal weight in a large population-based sample of adolescents in a country in the African region, using standardized measures of multiple components of physical fitness. We hypothesized that children who were overweight or obese would have lower fitness levels on all of the tests, especially the measure of cardiorespiratory fitness, the multistage shuttle run.

## Methods

### Setting

The study took place in the Republic of Seychelles, an archipelago located 1800 km east of Kenya in the Indian Ocean. The majority of the population is of African descent, with minorities of Caucasian, Indian, Chinese and mixed origins. The country has experienced rapid socio-economic development and the national gross domestic product per capita rose, in real terms, from US $2927 in 1980 to US $5239 in 2004. The country has experienced rapid health transition and the prevalence of overweight has increased markedly in both adults [19,20] and children [21]. Nearly 100% of students attend school up to the 10^th ^grade and 90% of them attend public schools.

### Participants and study design

All children attending secondary grades S1–S4 (7^th^–10^th ^school grades) in all public schools and in the one large private school performed 9 physical fitness tests in 2004, as part of a school-based national program for talent identification in sports. The fitness screening program was approved by the Ministry of Education and Youth and written consent for participation of the students was sought from the parents.

### Fitness tests

The fitness tests were conducted according to standardized protocols [22,23] and included the following nine tests: multistage shuttle run, 40-meter sprint, lateral jump, vertical jump, 5-meter shuttle run, small ball throw, basketball throw, sit ups, and push ups. These tests were performed at school during regular school hours by a team of 25 specially trained instructors over a 6-month period in 2004. The "multistage 20-meter shuttle run" is a validated measure of maximal oxygen uptake (VO_2_) [24] and consists of measuring the number of laps that are run back and forth between two lines set 20 meters apart at a running pace increased by 0.5 km per hour every minute using a pre-recorded audio tape. During the "5-meter shuttle test" children must change direction with two-foot stops between each shuttle: the test assesses agility and coordination. "Sit up" and "push up" tests assess muscular strength and endurance while "vertical" or "lateral jumps" tests assess lower body explosive power. Repeat tests were allowed for 40-meter sprint (2 trials), vertical jump (2) and lateral jump (3) and ball throws (2) and the best performance was recorded. Percentiles of fitness scores were calculated separately for each sex and 1-year age category.

### Anthropometric measurements

Weight and height were measured without shoes and body mass index (BMI) was calculated (kg/m^2^). "Overweight" and "obesity" were defined using the age- and sex-specific criteria of the International Obesity Task Force [25]. In order to categorize children with lowest weight and potentially lower muscular strength, we defined "lean" students as those having a BMI below the 10^th ^BMI percentile within each 1-year and sex category (hence 10% of students were "lean"). We refer to "normal" weight for students who are not "obese", "overweight" or "lean".

### Data analysis

Percentiles of performance were calculated for each of the nine fitness scores by sex and age. We examined the continuous relationship between fitness scores and both mean BMI and BMI percentiles using robust locally weighted regression, separately in boys and girls (LOWESS technique) [26]. The relationship between fitness scores and mean BMI allowed us to assess the shape of the relationship while the relationship between fitness scores and BMI percentiles allowed us to assess the impact of the relationship with regards to the actual distribution of BMI in the population. LOWESS regression allows to fit any relationship (i.e. any shape) between two variables but it cannot be adjusted for covariates. In order to control for age in these LOWESS analyses, we used mean BMI values, BMI percentiles, and fitness scores standardized to age 14, separately for boys and girls. Age standardization was based on a linear correction in view of the virtually straight relationship between age and both BMI and fitness scores in the considered four-year age range. Percentiles of BMI – determined by ranking BMI values – were determined based on age-standardized values of BMI. We calibrated, separately in boys and girls, the predicted fitness scores to range between 0 (poorest) and 1 (best) in order to enable direct comparison between results of the different fitness tests. In further analyses, we defined "good" performance as a fitness test passed with a result above the 75^th ^percentile, as calculated within each sex and 1-year age category (hence, 25% of boys and girls in each age category had "good" performance, overall) and we compared proportions of good performers (and 95% confidence intervals) across categories of body weight. These relationships were also examined by defining "good" performance based on fitness tests passed above the 90^th ^percentile. Analyses were performed with Stata 8.2.

## Results

The screening program was attended by 5420 of the eligible 5894 students in the four considered grades. This study was restricted to the 4599 students aged 12–15 years (1050, 1224, 1306, 1019 at ages 12, 13, 14 and 15, respectively) because other age categories included too few students for analyses (<250 per sex and 1-year age category). Data on all tests were available in 4343 students aged 12–15 (2202 boys, 2141 girls).

The prevalence of overweight (and not obesity) was 8.1% (95% confidence interval: 5.8–6.9) in boys and 13.1% (11.7–14.5) in girls, while the prevalence of obesity was respectively 3.1% (2.4–3.9) and 4.4% (3.5–5.3), consistent with findings in an independent other large school-based survey of overweight and obesity in children of Seychelles [21].

Table 1 shows the percentiles of performance for the nine fitness tests by sex and age. The results are ordered so that the highest percentiles correspond to the best performance. For each of the tests, performance was better in boys than in girls and fitness scores increased gradually with age in both boys and girls.

**Table 1:** Percentiles of performance for selected fitness tests by sex and age

			**Boys**									**Girls**								
				
**Age **(yr)	N	Centile	Agility [10*5 m runs] (sec)	Multi-stage shuttle run (n)	Sprint [40 m] (sec)	Jump lateral (cm)	Jump vertical (cm)	Throw small ball (m)	Throw basket ball (m)	Sit up in 30 sec (n)	Push up in 60 sec (n)	Agility [10*5 m runs] (sec)	Multi-stage shuttle run (n)	Sprint [40 m] (sec)	Jump lateral (cm)	Jump vertical (cm)	Throw small ball (m)	Throw basket ball (m)	Sit up in 30 sec (n)	Push up in 60 sec (n)
**12**	545 boys	P10	23.9	1.8	8.2	135	23	13.6	4.0	12	9	25.6	1.5	9.0	118	22	9.4	3.7	8	12
	505 girls	P25	22.8	2.8	7.6	150	26	16.6	4.5	15	14	24.3	1.8	8.3	129	25	11.1	4.1	11	15
		P50	21.7	4.0	7.2	166	30	19.8	5.0	18	18	23.0	3.0	7.7	144	28	12.8	4.7	14	19
		P75	20.7	5.8	6.8	182	33	22.9	5.6	21	23	21.8	4.0	7.2	160	31	15.1	5.1	17	23
		P90	19.9	7.0	6.3	198	38	26.0	6.3	24	28	20.9	5.3	6.8	176	35	17.5	5.6	19	27
		P95	19.4	7.5	6.1	207	40	28.0	6.9	26	31	20.3	6.3	6.5	185	37	19.0	6.0	21	29
**13**	631 boys	P10	23.7	2.0	8.0	140	26	15.5	4.5	13	10	25.5	1.5	9.0	118	23	10.0	4.0	8	11
	593 girls	P25	22.1	3.0	7.2	160	29	18.4	5.0	15	15	24.0	2.0	8.0	131	26	12.0	4.5	11	15
		P50	21.0	5.0	7.0	179	33	22.8	5.9	19	20	22.8	3.3	7.4	150	29	13.8	5.0	14	20
		P75	20.0	6.5	6.2	198	38	27.3	6.8	22	26	21.6	4.8	7.0	166	33	16.3	5.6	18	23
		P90	19.0	8.0	6.0	210	42	31.1	7.7	25	30	20.7	5.8	6.4	182	36	19.2	6.0	21	28
		P95	18.6	8.3	5.8	220	45	34.0	8.0	26	33	20.0	6.3	6.0	192	39	21.0	6.3	22	30
**14**	658 boys	P10	23.0	2.5	8.0	150	27	18.0	5.0	14	11	25.0	5.8	8.6	185	37	20.6	6.2	21	29
	648 girls	P25	21.5	4.0	7.0	172	31	22.0	6.0	17	16	23.7	4.8	8.0	170	33	17.5	6.0	18	25
		P50	20.2	5.8	6.5	196	36	26.4	6.8	20	21	22.0	3.8	7.2	151	30	15.0	5.3	15	21
		P75	19.4	7.0	6.0	211	40	31.7	7.6	23	28	21.0	2.3	6.9	135	27	12.2	4.9	12	17
		P90	18.8	8.0	5.8	228	44	36.5	8.5	26	33	20.2	2.0	6.4	119	24	10.7	4.3	9	14
		P95	18.3	8.8	5.6	236	47	39.0	9.0	28	38	19.9	1.5	6.0	113	22	9.9	4.0	6	11
**15**	503 boys	P10	22.7	3.5	7.2	165	30	20.0	6.0	15	13	25.1	2.0	8.8	120	24	11.0	4.5	9	12
	516 girls	P25	21.2	5.0	6.9	190	34	25.1	6.6	18	17	24.0	2.8	8.0	138	27	13.0	5.0	11	16
		P50	20.0	6.5	6.1	208	39	30.2	7.6	21	22	22.5	4.0	7.3	154	30	15.1	5.4	15	20
		P75	19.1	8.0	6.0	225	43	35.6	8.3	24	28	21.2	5.0	7.0	172	34	18.3	6.0	18	25
		P90	18.6	8.6	5.7	241	47	39.8	9.0	27	35	20.4	6.0	6.4	193	37	22.0	6.4	20	30
		P95	18.0	9.2	5.4	247	49	42.6	9.6	28	40	20.0	6.5	6.0	200	39	24.1	6.8	23	34

**Figure 1:**
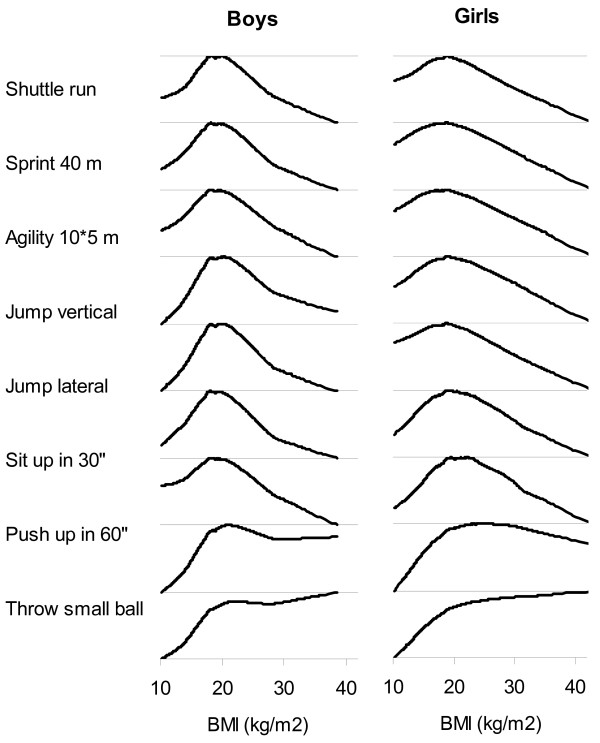
Locally weighted regression between fitness scores and body mass index for 9 fitness tests in 2202 boys and 2141 girls aged 12–15 years. Body mass index and fitness scores are standardized for age and fitness scores are calibrated to range from 0 (lowest performance) to 1 (highest performance).

Figure 1 shows that for all tests except the ball throws, the relationship between fitness scores and BMI followed an inverse graded J-shape: fitness results were best among students with "normal" BMI, lower among "lean" students and lowest among students with highest BMI. However, students with highest BMI performed as well as or better than students with normal BMI for both the small ball and basketball throws. The shape of the relation between fitness performance and BMI was fairly similar for all tests except for the ball throws (i.e. for all tests that are sensitive to both muscular strength and body weight). To control for the effect of age, both the fitness scores and BMI were standardized for age.

**Figure 2:**
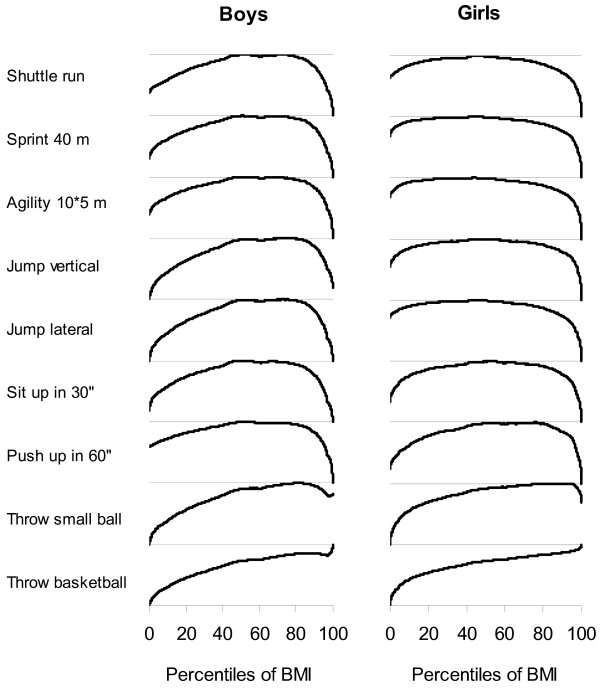
Locally weighted regression between fitness scores and percentiles of body mass index for 9 fitness tests in 2202 boys and 2141 girls aged 12–15 years. Body mass index and fitness scores are standardized for age and fitness scores are calibrated to range from 0 (lowest performance) to 1 (highest performance).

Figure 2 shows the relationship between fitness scores and BMI percentiles. Both the fitness scores and the BMI percentiles are standardized for age. For all tests, except the ball throws, mean fitness scores were highest in the large proportions of students with "normal" weight. Mean fitness scores, except the ball throws, were lower in the small proportions of students with the highest BMI percentiles (mostly the 10% of children with BMI percentiles larger than 90). Mean fitness scores (except the ball throws) were also lower among children with the lowest BMI (e.g. among the 20–30% of children who had BMI percentiles lower than 70–80). The trends toward lower fitness scores in the leanest children was more marked in boys than in girls. This sex difference was not related to lower absolute fitness scores in girls than in boys since fitness scores were calibrated from 0 to 1 separately in boys and girls.

Table 2 shows the proportions of boys and girls who passed the fitness tests above the 75^th ^percentile and above the 90^th ^percentile, by category of body weight. Cut off values for BMI categories (lean, normal, overweight and obese) and for all fitness scores were calculated separately for boys and girls and for each 1-year age category, which removes potential confounding effects of age or sex. For all tests except the ball throws, the proportion of children with good performance was lower among overweight and obese children as compared to children with normal weight. This pattern was found in both boys and girls and whether performance was assessed on the basis of a fitness test passed above the 75^th ^percentile or above the 90^th ^percentile. The proportion of boys with good performance was also lower in lean students than normal weight students for all tests. The difference in fitness performance between lean and normal weight children was less marked among girls. For the ball throws, the proportion of children who achieved good performance increased gradually across lean, normal, overweight and obese categories.

**Table 2:** Proportion (in percent) of adolescents who passed fitness tests with a result above the 75^th ^percentile (P75) or above the 90^th ^percentile (P90), by body weight status.

	**Boys**						**Girls**					
		
	**>P75**			**>P90**			**>P75**			**>P90**		
**Shuttle run**												
Lean	19	(14–24)	*	8	(5–12)	*	32	(26–38)		14	(9–18)	
Normal	30	(28–32)		15	(13–17)		29	(27–32)		11	(10–13)	
Overweight	8	(4–12)	*	3	(0–5)	*	8	(5–11)	*	1	(0–3)	*
Obese	1	(0–4)	*	0		*	1	(0–3)	*	0		*
**Sprint 40 m**												
Lean	21	(15–26)	*	6	(2–9)	*	36	(30–42)		13	(8–17)	
Normal	37	(35–39)		16	(14–17)		37	(35–40)		12	(10–13)	
Overweight	11	(6–15)	*	2	(0–4)	*	20	(15–25)	*	3	(1–5)	*
Obese	1	(0–4)	*	0		*	3	(0–7)	*	0		*
Agility 10*5 m												
Lean	19	(14–25)	*	8	(4–11)	*	29	(23–35)		7	(4–10)	*
Normal	32	(30–35)		13	(11–15)		29	(27–31)		13	(11–14)	
Overweight	6	(3–10)	*	2	(0–4)	*	14	(10–18)	*	3	(1–5)	*
Obese	3	(0–7)	*	0		*	4	(0–8)	*	1	(0–3)	*
**Jump vertical**												
Lean	18	(13–24)	*	4	(1–7)	*	25	(19–31)		12	(7–16)	
Normal	32	(30–34)		13	(11–15)		33	(30–35)		14	(12–15)	
Overweight	12	(7–17)	*	5	(1–8)	*	19	(14–23)	*	7	(4–10)	*
Obese	0		*	0		*	7	(2–13)	*	4	(0–8)	*
**Jump lateral**												
Lean	10	(6–14)	*	2	(0–4)	*	33	(27–39)		12	(7–16)	
Normal	31	(29–34)		13	(11–14)		30	(28–32)		13	(11–14)	
Overweight	7	(3–11)	*	3	(1–6)	*	10	(6–13)	*	3	(1–5)	*
Obese	1	(0–4)	*	0		*	1	(0–3)	*	0		*
**Sit up in 30"**												
Lean	24	(18–29)	*	9	(5–13)		27	(21–33)		11	(7–15)	
Normal	34	(31–36)		13	(11–14)		31	(28–33)		14	(12–16)	
Overweight	11	(7–16)	*	6	(3–10)	*	21	(17–26)	*	8	(5–11)	*
Obese	7	(1–13)	*	3	(0–7)	*	11	(4–17)	*	4	(0–8)	*
**Push up in 60"**												
Lean	21	(16–27)	*	7	(4–10)	*	29	(23–35)		10	(6–14)	
Normal	30	(28–32)		13	(12–15)		31	(29–33)		12	(10–14)	
Overweight	7	(3–11)	*	2	(0–4)	*	26	(21–31)	*	10	(6–14)	*
Obese	6	(0–11)	*	1	(0–4)	*	15	(8–22)	*	7	(2–13)	*
**Throw small ball**												
Lean	4	(1–7)	*	0		*	11	(7–15)	*	3	(1–5)	*
Normal	28	(26–30)		12	(10–13)		27	(25–29)		11	(10–13)	
Overweight	25	(19–32)		10	(5–14)		28	(23–33)		10	(7–13)	
Obese	16	(7–25)	*	3	(0–7)	*	30	(20–39)		11	(4–17)	
**Throw basketball**												
Lean	3	(1–5)	*	0		*	5	(2–8)	*	1	(0–2)	*
Normal	27	(25–29)		11	(9–12)		25	(22–27)		10	(9–12)	
Overweight	33	(26–40)		20	(14–26)	*	43	(37–49)	*	23	(18–27)	*
Obese	46	(35–58)	*	25	(14–35)	*	50	(40–60)	*	21	(13–20)	*

BMI and fitness scores are standardized for age.

"Lean" refers to children with age and sex specific BMI <10^th ^percentile.

95% confidence intervals are indicated between brackets.

* refers to statistically significant difference in comparison to children with "normal" weight (*p *< 0.05).

**Figure 3:**
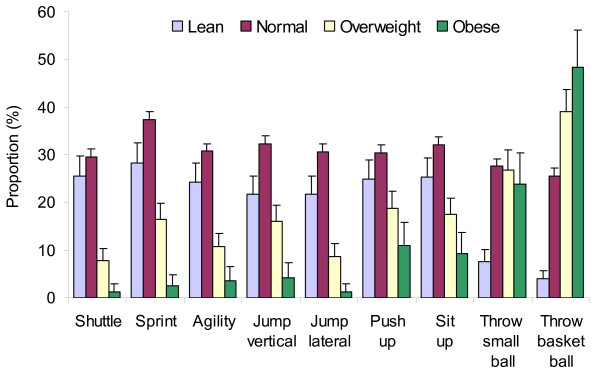
Proportion (and upper 95% confidence interval) of students performing physical fitness tests above the 75^th ^percentile, by body weight categories (n = 4599). Cut off values for good performance and for body weight categories were calculated separately for boys and girls and for each 1-year age categories. Difference between obese or overweight vs. normal weight is statistically significant for all tests except for small ball throw; difference between lean weight and normal weight is significant for sprint, agility, jumps, sit up, and ball throws.

Figure 3 illustrates the proportions of all children (boys and girls) who passed the fitness tests above the 75^th ^percentile, comparing children in lean, normal, overweight and obese categories. For all tests, except the ball throws, the proportion of students with "good" performance was markedly lower among overweight or obese students than among students with normal weight (*p *< 0.05). For example, 29% of students with normal weight had good performance on the multistage shuttle run vs. 8% of overweight students and 1% of obese students. However, for the basketball throw (a test that does not rely on body lifting or moving), overweight or obese students had good performance more often than lean students (*p *< 0.05). Lean students had good performance less often than children with "normal" weight for several tests, although the difference was weaker than the difference between normal and overweight/obese students. Lean students performed particularly poorly for the ball throws. Analysis performed separately in boys and in girls showed similar results.

## Discussion

In this nationally representative sample of over 4000 adolescents aged 12–15 in the Republic of Seychelles, we found a strong inverse relation between excess body weight and physical fitness, using direct measurement for weight status and standardized physical fitness tests. Fitness scores were also lower in children with lowest BMI.

The association between fitness and excess body weight was particularly marked for tests involving agility and mobility (multistage shuttle run, 40-meter sprint, 5-meter shuttle run test) as compared to tests relying more on strength (sit-up and push up tests). Findings for the multistage shuttle run are of particular interest as this test has been validated to assess cardiovascular fitness (24). The better performance of overweight children for the basketball throw reflects a test that requires mainly strength and is insensitive to body weight.

There was a trend toward lower performance in lean students, as compared to students with normal weight for all fitness tests. This trend is unlikely related to illness in view of the large proportions of adolescents involved (e.g. as many as 20–30% of boys in the lower tail of the BMI distribution displayed markedly lower performance, as appears in Figure 2). The trend toward lower performance in lean students, as compared to students with normal weight, is likely consistent with a proportionate ratio of muscular mass to total body weight that may be less favorable in lean than average weight children. Fitness tests indeed evaluate a combination of muscle strength (favoring children with normal weight over lean children) and leanness (favoring children with normal weight over heavy children).

Physical fitness is a function of both physical activity and non-modifiable factors such as genetics [27]. Because we did not measure physical activity and these other factors, we cannot determine their relative contribution. In addition, the cross-sectional design of the study does not permit us to distinguish whether low fitness precedes or follows excess body weight. However, the strong inverse association between high fitness and excess body weight is compatible with lower physical activity in obese children and decreased energy expenditure as a possible determinant of obesity in this sample of adolescents. Longitudinal studies are needed to determine the directionality of the relationship between fitness and obesity. Furthermore, we did not have a direct measure of body composition in this study. Future studies examining the relationship between adiposity and fitness should consider using such a measure to provide a more accurate assessment.

An association between low fitness and several detrimental metabolic outcomes has been well documented in children [5-7]. It is yet unclear whether improved health outcomes in children who are fit are mediated through leaner body weight [28] or through other mechanisms [29]. Other mechanisms may include increased insulin sensitivity, a non-insulin-dependent glucose uptake that causes lower insulin release, an improved ratio between HDL cholesterol and LDL cholesterol because of increased activity of lipoprotein lipase, or improved function of other metabolic hormones and enzymes related to fat metabolism [29]. Furthermore, low fitness seems to be associated with lesser subsequent obesity in adults [30], a further benefit of fitness that extends into adulthood.

Therefore, because low fitness is associated with adverse health outcomes, there is an interest in addressing low fitness, most likely through increasing physical activity. However, because obese children in this study tended to be unfit, such physical activity interventions should recognize this relationship and a special approach might be needed that is responsive to the ability and needs of overweight children.

It is possible that the lower physical fitness associated with excess body weight may be a barrier impeding physical activity and training differentially among students with or without excess body weight. For example, obese children may be less likely to engage in physical activity because of fear of poor performance and stigmatization. Such a barrier to participation in sports and physical activity could also be a mechanism likely to perpetuate overweight/obese status. This may underlie the need for specific technical, psychological and social support to encourage participation of overweight youth in sports at school and in other settings.

## Conclusion

This large population-based survey shows a strong inverse relationship between overweight and several standardized tests of physical fitness in adolescents in a country in the African region. This inverse association is compatible with lower physical activity as a possible determinant of obesity in this sample of adolescents. Since low physical fitness is associated with increased cardiometabolic risk and with tracking into adulthood, the findings provide further support to implement interventions to reduce overweight in youth and to promote physical exercise as a means to achieve physical fitness. Further research should examine the role of overweight and/or low physical fitness as factors limiting the participation of youth in physical activities and sports and identify strategies and interventions that could overcome such potential barriers.

## Competing interests

The author(s) declare that they have no competing interests.

## Authors' contributions

PB analyzed the data and led the write-up of the paper. RA had a key role for organizing and supervising the project and reviewed the paper. HB collaborated to the write-up of the paper. All authors read and approved the final manuscript.
